# Passive and Dynamic Stabilizers of First Ray Alignment After TMT-I Fusion: The C1-C2 Intercuneiform Ligament and Peroneus Longus in a Sequential Cadaveric Destabilization Model

**DOI:** 10.1177/10711007261460458

**Published:** 2026-07-21

**Authors:** Alena Richter, Peter Savov, Sarah Ettinger, Leif Claassen, Daiwei Yao, Frederik Emanuel Mann, Anna Altemeier, Christian Plaass

**Affiliations:** 1 Orthopeadic Clinic of Hannover Medical School, Diakovere Annastift, Germany; 2 Division of Orthopeadics at Campus Pius-Hospital, School of Medicine and Health Sciences Carl von Ossietzky Universität Oldenburg, Germany; 3 Orthoprofis Hannover, Germany; 4 Fachärzte Rhein-Main, Seligenstadt, Germany; 5 Klinik für Plastische Chirurgie, Helios-Klinikum Berlin-Buch, Germany

**Keywords:** hallux valgus, tarsometatarsal instability, tarsometatarsal fusion, hallux valgus recurrency, TMT-I

## Abstract

**Background:**

Current guidelines recommend first tarsometatarsal joint (TMT-I) fusion in case of symptomatic hallux valgus (HV) with TMT-I instability. Despite successful TMT-I fusion, recurrence rates of 1.7% to 2.9% have been reported suggesting that residual instability of surrounding structures may contribute to recurrent deformity. The aim of this study was to measure the effect of sequential sectioning of the surrounding ligamentous structures in a TMT-I fusion model with and without activation of the peroneus longus tendon.

**Methods:**

Crossed-screw TMT-I fusion was performed in 6 cadaveric feet, and an optical infrared marker camera system was installed to detect relative movements between the first and second metatarsal head. A transverse force of 10 N and a vertical force in the coronal plane of 15 N were applied on the first metatarsal head and sectioning of the M. abductor hallucis, the dorsal and plantar Lisfranc ligaments, and the medial-middle intercuneiform ligament (C1-C2) were performed. The influence of the peroneus longus tendon (PL) was additionally evaluated.

**Results:**

In the transverse plane, significant changes occurred after sectioning of dorsal and plantar Lisfranc ligaments and predominantly after cutting the medial-middle intercuneiform ligament (C1-C2) without PL force (mean difference: 2.3°, 95% CI: −4.16 to −0.4°, *P* = .0234). In coronal plane, the overall effect of ligament release was not significant (*P* = .132); post hoc analysis identified a significant increase in first metatarsal translation only after release of the M. abductor hallucis tendon without PL force (mean difference: 0.9°, 95% CI: −1.68 to −0.09°, *P* = .033). There was a significant influence of the PL force, especially in transverse plane (*P* < .001).

**Conclusion:**

Sequential sectioning of the M. abductor hallucis, dorsal and plantar tarsometatarsal ligaments, and the medial-middle intercuneiform ligament was associated with progressive first ray instability in this cadaveric model. PL activation provided a significant dynamic stabilizing effect, particularly in the transverse plane, where it compensated for progressive ligamentous insufficiency; in the coronal plane, abductor hallucis release was the primary destabilizing event and PL compensation was significant only under full destabilization.

## Clinical Relevance Statement

The findings suggest that the C1-C2 intercuneiform ligament is an important passive restraint against transverse first ray displacement after TMT-I fusion, and that peroneus longus activation may compensate for progressive ligamentous insufficiency, particularly in the transverse plane. They provide a biomechanical rationale for intraoperative assessment of intercuneiform stability and for future studies examining whether additional fixation or peroneus longus rehabilitation can reduce recurrence after TMT-I fusion.

## Introduction

Hallux valgus (HV) is one of the most common forefoot deformities, characterized by lateral deviation of the great toe and medial deviation and pronation of the first metatarsal bone, leading to increasing subluxation of the sesamoids.^1,2^

Morton^3^ primarily developed the concept of medial column instability leading to foot pathologies and clinical dysfunction. Klaue et al^4^ transferred the concept to HV focusing on dorso-plantar instability of the first tarsometatarsal joint (TMT-I) as central mechanism. Instead of a straight dorso-plantar instability, a plane of 45° from plantar-lateral to dorsal-medial has been described by Singh et al^5^ referring to the physiological pronation of the foot with dorso-medial shifting of the first metatarsal during gait.

Although a restoration of tarsometatarsal stability after first ray realignment without stabilizing procedures of the TMT-I has been shown,^6,7^ an overall recurrence rate of 4.9% in the short term and rates up to 30% to 70% in the long-term after osteotomy techniques are described.^8,9^ Therefore, arthrodesis of the TMT-I joint is favoured by many surgeons for severe HV and clinically TMT-I instability^10^ showing a recurrence rate of 1.7% to 2.9%.^8,11^

Nevertheless, Fleming et al^12^ also noticed a high rate of intermetatarsal instability in the transverse plane during surgical HV correction describing a rather mutiplanar tarsometatarsal instability. Earlier, Lapidus suggested a medial-middle intercuneiform fusion in case of severe intermetatarsal instability,^13^ showing good clinical results with a higher stability, but a subjective feeling of stiffness and an increased risk of complications are described.^14-17^

Currently, first ray hypermobility in the pathogenesis of HV is highly discussed.^6-9,11,18,19^ In recurrent HV, a high incidence of tarsometatarsal instability of up to 96% has been found.^20^ Both osseous characteristics and muscle and ligament function contribute to first ray stability.^21^ So far, the relevance of the different stabilizing structures of the midfoot before and after TMT-I fusion are still controversial.

The stabilizing effect of the peroneus longus tendon is a result of its plantarflexing, everting, and lateralizing force on the first metatarsal.^22^ The effect of the peroneus longus tendon on first ray alignment has also been shown to be preserved after TMT-I arthrodesis.^23,24^ As shown in Lisfranc injuries, first ray stability is maintained by the dorsal and plantar tarsometatarsal ligaments, the interosseous Lisfranc ligament from the medial cuneiform to the base of the second metatarsal and the medial-middle intercuneiform ligament.^25,26^

Although the stabilizing effect of the peroneus longus tendon on first ray alignment is preserved and clinically relevant after TMT-I arthrodesis, the role of tarsometatarsal ligaments in recurrent medial column malalignment after TMT-I arthrodesis is unclear.

The aim of this study was to measure the effect of sequential sectioning of the surrounding ligamentous structures in a TMT-I fusion model with and without activation of the peroneus longus tendon.

## Material and Methods

### Cadaver Preparation

Six cadaveric feet (Science Care) were analyzed in this biomechanical study, which is in line with sample sizes reported for similar biomechanical cadaver experiments on first ray and tarsometatarsal biomechanics.^27^ None of the specimens showed any deformities or previous surgeries. The mean age of the donors was 75.5 years (range 50-90 years). Two donors were female. All fresh frozen feet were stored at −23 °C and thawed the day before the experiment. The tibiae were resected 15 cm above the upper ankle and embedded in a 2-component polyurethane casting resin at the proximal ends (Rencast FC52/53; Huntsman Corp). The tendons of the M. tibialis posterior (TP), M. tibialis anterior (TA), M. peroneus longus (PL), and M. peroneus brevis (PB) were prepared and fixed with one clamp each. Further, the dermis and cutis were prepared while sparing the retinaculum extensorum inferius, the C1-C2 intercuneiform ligament, and the capsule of the TMT-I as well as the M. abductor hallucis. The arthrodesis of the TMT-I was performed using 2 crossed screws in order to simplify the biomechanical setup. Intraoperative fluoroscopy and visual inspection under axial loading were used to confirm adequate fusion construct stability throughout the experiment.

### Measurement System

An active optical infrared LED marker camera system, the NDI Optotrak Certus System, was used to optically record the relative movements of the metatarsal (MT) and tarsal bones with a resolution of 0.01 mm. A total of 4 measurement points were established to create a 3-dimensional coordinate system:

1. Reference point: Os naviculare—central placement
2. First metatarsal—central diaphyseal placement
3. Second metatarsal—central diaphyseal placement
4. Os cuneiforme mediale—central placement for relative cuneiform motion

**Figure 1.**
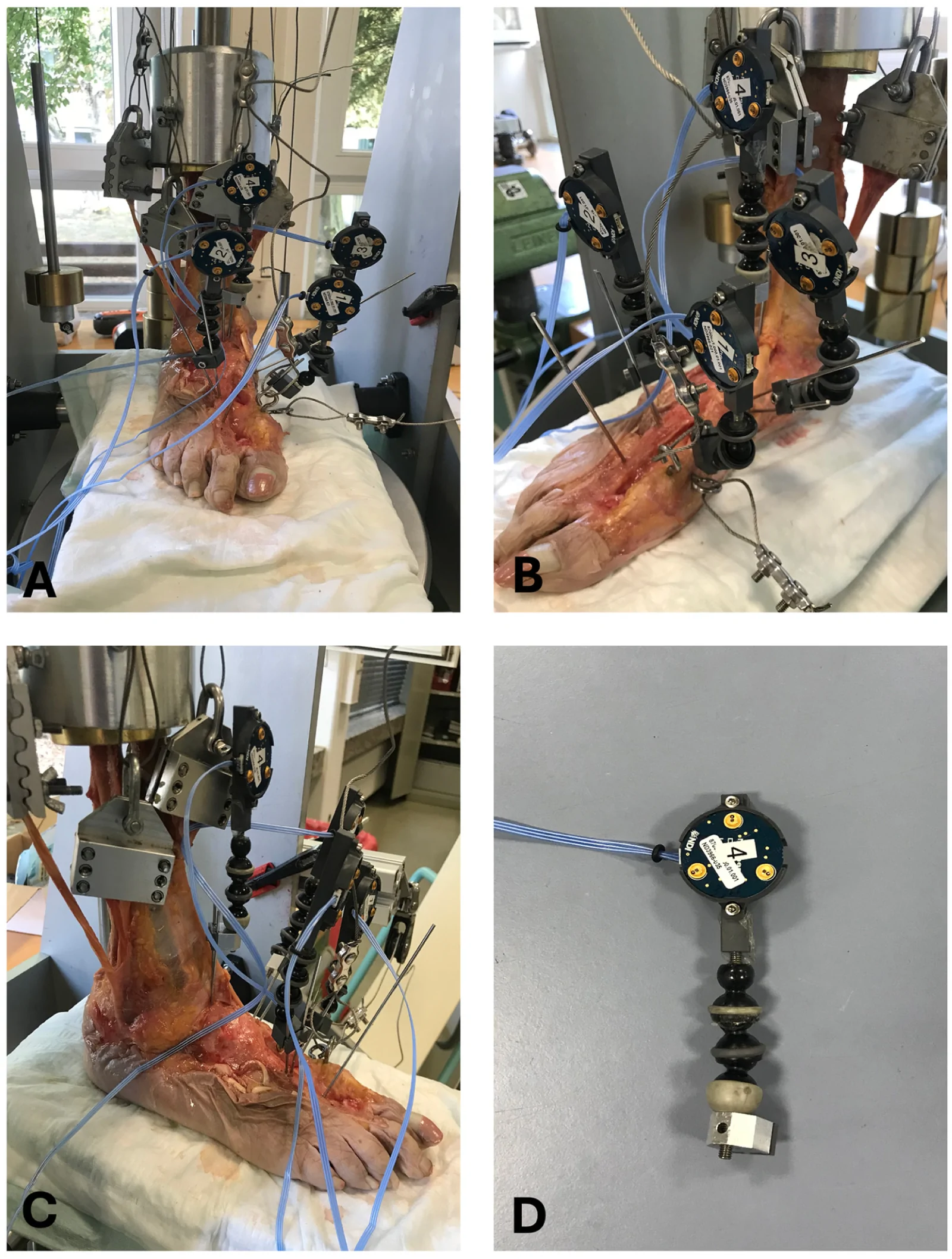
Experimental setup in the custom forefoot simulator with axial loading and tendon traction (A-C). Four tripod-mounted infrared marker clusters (GorillaPod–Rigid Body complexes) are attached to the navicular, first and second metatarsals, and medial cuneiform using Kirschner wires for 3-dimensional motion tracking. Panel D shows one constructed tripod with its infrared marker cluster as an example.

Three infrared markers were installed at each measurement point. These infrared markers were firmly anchored to a rigid body (RB). The RB was in turn attached to a constructed GorillaPod base, a flexible tripod base that conforms to curved bone surfaces (JOBY, GorillaPOD 325) using a lag screw. This GorillaPod–Rigid Body Complex (GP-RBK) was then fixed to the specific measurement points of the forefoot using 2 Kirschner wires. All GP-RBKs were connected to the Optotrak system via electrodes. The measurements of the relative movements during the experiments were recorded via dedicated software from NDI. The experimental setup is shown in Figure 1.

**Figure 2.**
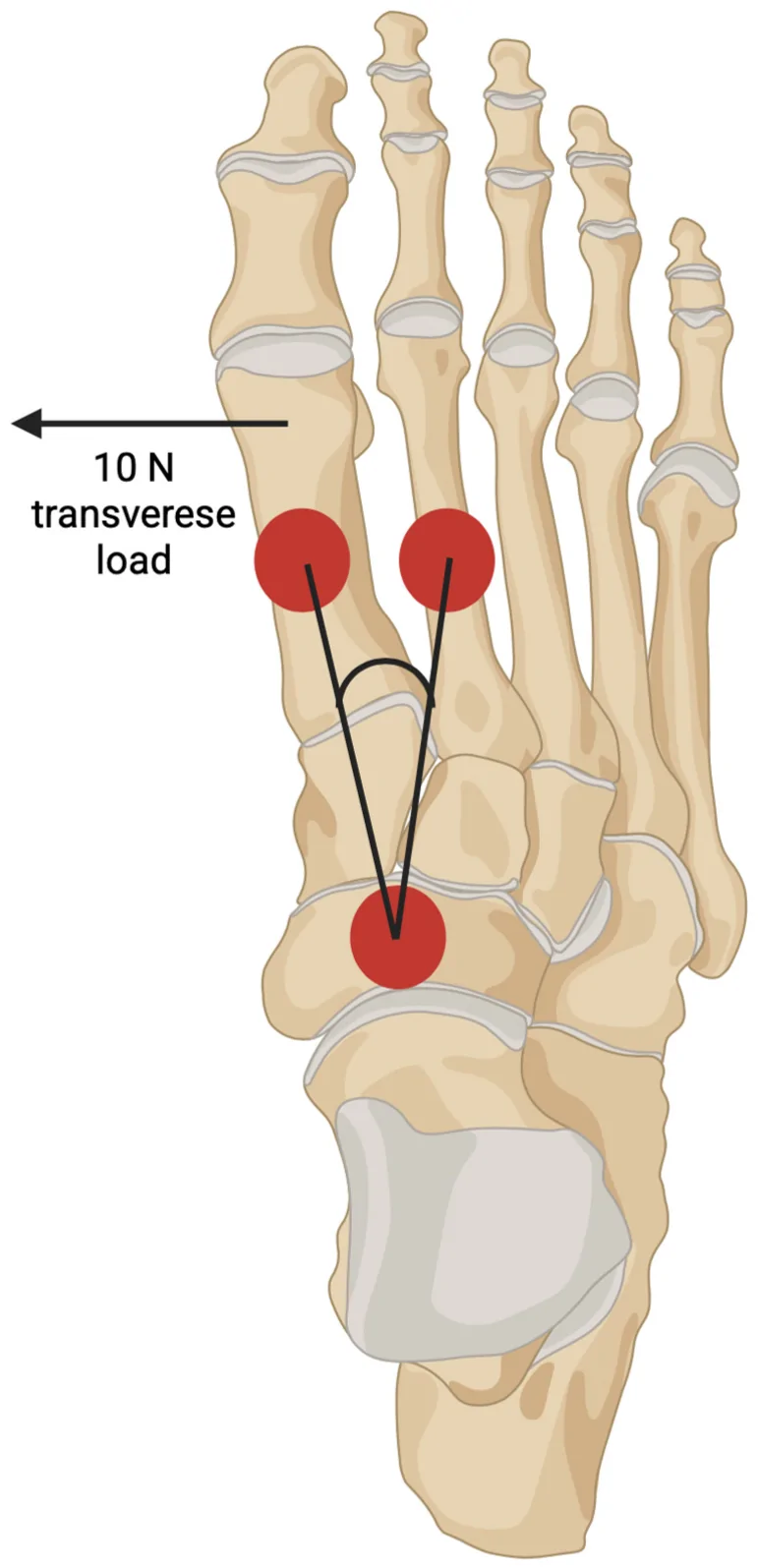
Schematic illustration of the experimental setup, marker configuration, and load application in transverse plane. A transverse load of 10 N was applied to the distal forefoot in lateral direction. Red markers indicate infrared markers attached to the first and second metatarsal shafts and to the navicular bone. The angle between the vectors connecting the navicular marker with the markers on metatarsals I and II was calculated and used as a measure of transverse forefoot angular displacement relative to the navicular reference point. Created in BioRender. Richter, A. (2026) https://BioRender.com/nww3pwy. [See online article for color figure.]

A relative angle measurement was made in the X, Y, and Z axes. The coordinate system was defined as follows: the X axis represented superior-inferior motion (coronal plane), the Y axis represented mediolateral motion (transverse/horizontal plane), and the Z axis represented anterior-posterior motion (sagittal plane). Angular displacement was calculated as the change in the angular relationship between the MT-I and MT-II rigid body coordinate systems relative to the navicular reference frame. Additionally, a marker was placed on the medial cuneiform. Following arthrodesis of the TMT-I, the medial cuneiform is rigidly connected to the first metatarsal but remains proximally articulated with the navicular bone, thereby allowing optional assessment of relative motion at the cuneonavicular joint. The corresponding marker trajectories were not further analyzed in the present study because stability of the first tarsometatarsal fusion was confirmed radiographically, and quantitative assessment of relative motion at this joint was therefore beyond the primary scope of the investigation. A schematic of the testing setup, marker location, and force application is depicted in Figure 2.

### Force Application System

The specimens were placed in a special custom-made frame that provides an accurate alignment of the feet in neutral position with the heel on the ground and an axial loading of the tibia, with 200 N representing weight bearing. All tendons were loaded with weights using clamps and a pulley (Figure 1). According to other biomechanical studies in the literature, the tendons of the PB and the TP were loaded with 5 N. The tendon of the TA was loaded with 15 N. In contrast, the PL tendon was loaded with different weights during the experimental procedure (0 and 10 N). For the application of vertical and transverse traction forces to the first metatarsal head, a 6 × 66-mm lag screw was inserted into MT-I and connected to steel cables that were loaded with defined weights via pulleys and guide rails within the forefoot simulator (Figure 1).

The procedure of the experiment was standardized and carried out in exactly the same order in each case. The basic element of the experiment was a vertical traction in the coronal plane of 15 N and a transverse traction of 10 N on the MT-I head with regard to translation compared to the second metatarsal head.

The transverse load of 10 N and vertical load of 15 N were selected as a subphysiologic but realistic magnitude within the range of intermetatarsal forces reported between the first and second metatarsals in cadaveric models and without approaching the higher axial loads typically used to simulate full weight-bearing in cadaveric midfoot models, in order to avoid failure while still challenging the stabilizing structures.^28,29^

**Figure 3.**
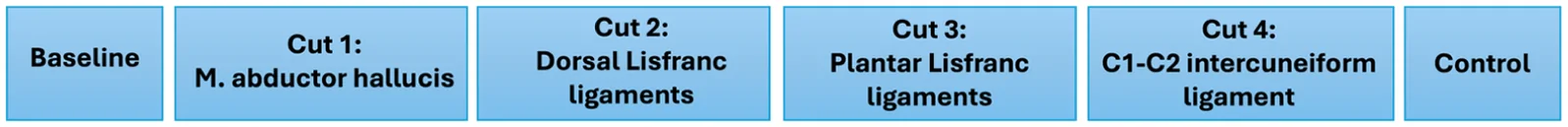
Schematic overview of the experimental conditions: baseline, sequential release of the abductor hallucis (cut 1), dorsal Lisfranc ligaments (cut 2), plantar Lisfranc ligaments (cut 3), and C1-C2 intercuneiform ligament (cut 4), followed by a final control condition with complete destabilization of the TMT‑I row soft tissue connections were released to confirm experimental setup integrity (cut 5).

Furthermore, the influence of the PL tendon was investigated by varying the traction. Each part of the experimental procedure was performed with 0-N and 10-N PL traction. The experimental procedure included baseline measurement, 4 levels of destabilization, and a control measurement at the end. In the first cut, the M. abductor hallucis was transected at its insertion at the joint capsule of the first MTP joint. The tendon of the abductor hallucis was preserved but not loaded via weights; in this model, it was therefore considered a passive static stabilizer rather than an actively loaded structure. In the following levels, the dorsal Lisfranc ligaments (cut 2), the plantar Lisfranc ligaments (cut 3), and the C1-C2 intercuneiform ligament (cut 4) and all other ligaments of the TMT-I row, including the residual capsular ligaments of the TMT-I and soft tissue connections between the bases of the first and second metatarsals (control), were cut. Each level contains 6 steps, which are illustrated in detail in Figure 3.

### Parameters

Translation was measured as angular displacement in degrees between the MT-I and MT-II coordinate systems. This angular measurement represents the relative rotational change between the 2 metatarsal heads when forces are applied quantifying how much MT-I moves medially (transverse plane) or dorsally (coronal plane) relative to MT-II. The baseline value of the translation (step 1 and 2 in baseline measurement) was subtracted from the respective measured values of the other steps. Control measurement is not included in the calculations of the statistical evaluation, because it is only used to check the experimental setup.

### Statistical analysis

One-way analysis of variance (ANOVA) was performed to calculate significance in each test series after the destabilization levels. Furthermore, Sidak’s multiple comparisons test was performed for subanalysis to calculate significant differences between each section. The *P* value was adjusted. This was done separately for the transverse and coronal traction measurements. To evaluate the influence of peroneal traction on stability, a 2-way ANOVA analysis was performed. GraphPad Prism 7.0 was used to perform the statistical analysis.

## Results

### Transverse Force

**Figure 4.**
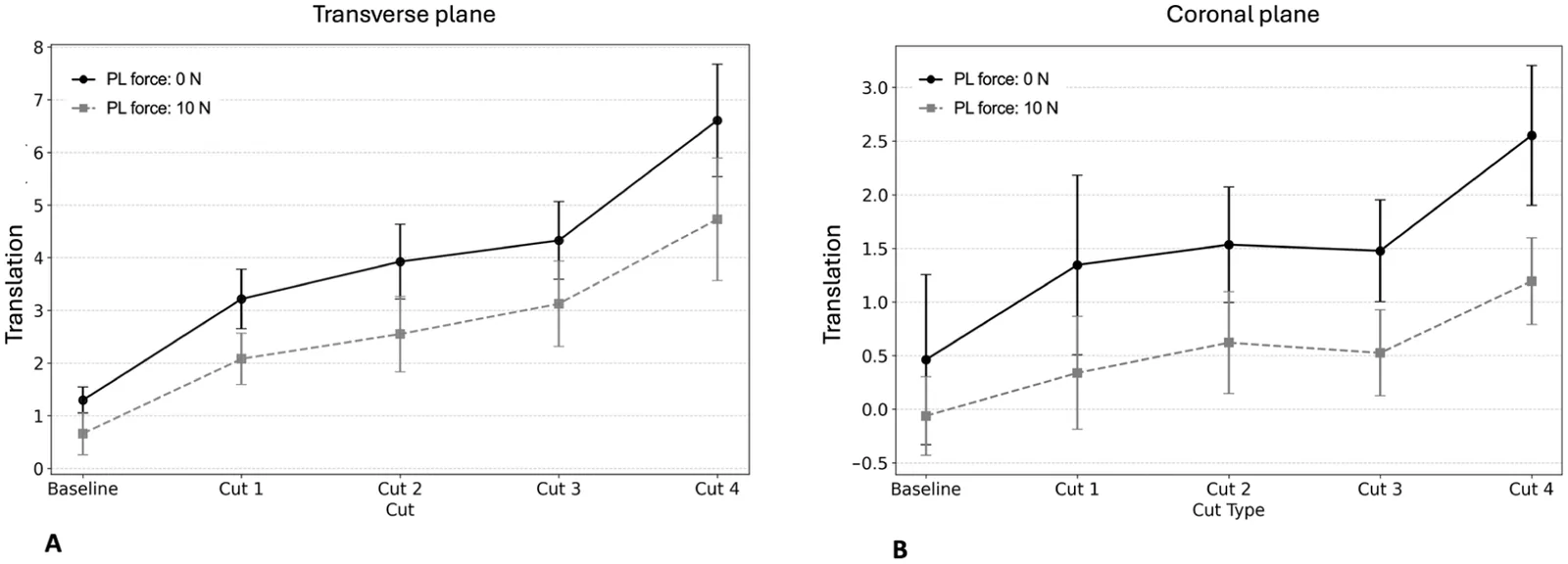
Angular displacement (degrees) of the MT-I head compared with the MT-II head, referenced to the navicular coordinate frame, with 0-N and 10-N peroneus longus force across destabilization levels. (A) Transverse plane: 10-N lateral traction applied to MT-I head. (B) Coronal plane: 15-N vertical traction applied to MT-I head. MT, metatarsal.

The translation of the MT-I head compared to the MT-II head after transverse traction with 0-N and 10-N PL force during the destabilization levels is shown in Figure 4. The 1-way ANOVA demonstrated a significant overall effect of ligament release for both PL conditions (0-N PL force, *P* = .0022; 10-N PL force, *P* = .0038). Significant changes between destabilization steps were observed after cut 2 (dorsal Lisfranc ligaments) (*P* = .0355) and cut 3 (plantar Lisfranc ligaments), with the largest increase after cutting the C1-C2 intercuneiform ligament (cut 4) under 0-N PL force (*P* = .0234) (Table 1). Under 10-N PL force, no significance was detected in the Sidak’s multiple comparisons analysis. Correspondingly, the 2-way ANOVA analysis shows a significant influence of the PL force (*P* value < .001) (Table 2).

**Table 1.** Sidak’s Multiple Comparisons Test for Transverse Traction of the First Metatarsal Head (0-N vs 10-N PL Force Analyzed Separately).^a^

Comparison	0-N PL Force			10-N PL Force		
	Mean Difference (degrees)	Adjusted *P* value	95% CI (degrees)	Mean Difference (degrees)	Adjusted *P* Value	95% CI (degrees)
Baseline vs Cut 1	1.9 ± 0.62	.1057	−4.3 to 0.45	1.4 ± 0.40	.065	−2.95 to 0.1
Cut 1 vs 2	0.7 ± 0.17	.0355*	−1.36 to −0.06	0.5 ± 0.28	.481	−1.52 to 0.58
Cut 2 vs 3	0.4 ± 0.08	.0163*	−0.71 to −0.1	0.6 ± 0.27	.298	−1.59 to 0.44
Cut 3 vs 4	2.3 ± 0.50	.0234*	−4.16 to −0.4	1.6 ± 0.56	.137	−3.74 to 0.53

a Values are mean differences in angular translation (degrees) between destabilization levels ± SE. The 95% CIs relate to the mean difference between the 2 levels being compared and do not refer to the absolute mean translation at each level.

* Significance.

**Table 2.** Two-Way ANOVA and Sidak’s Multiple Comparisons Test for the Effect of Peroneus Longus (PL) Force During Transverse Traction of the First Metatarsal Head.^a^

	0-N PL Force	10-N PL Force			
	Mean Translation (degrees)	Mean Translation (degrees)	Mean Difference ± SE (degrees)	Adjusted *P* Value	95% CI
Baseline	1.3	0.7	0.6 ± 0.38	.495	−0.28 to 1.55
Cut 1	3.2	2.1	1.1 ± 0.38	.045	0.22 to 2.05
Cut 2	3.9	2.6	1.4 ± 0.38	.011	0.46 to 2.29
Cut 3	4.3	3.1	1.2 ± 0.38	.031	0.29 to 2.12
Cut 4	6.6	4.7	1.9 ± 0.38	<.001*	0.96 to 2.79

a The columns “0-N PL Force” and “10-N PL Force” show the mean angular translation (degrees) at each destabilization level. The column “mean difference ± SE” gives the mean difference in translation between 0-N and 10-N PL force (0 N minus 10 N) ± SE, followed by the 95% CI of this mean difference.

* Significance.

### Vertical Force in Coronal Plane

The translation of the MT-I head compared to the MT-II head after vertical traction in the coronal plane with 0-N and 10-N PL force is shown in Figure 4. Translation in the coronal plane remained low throughout the destabilization sequence. The 1-way ANOVA showed no significant overall effect of ligament release for either PL condition (0 N: *P* = .132; 10 N: *P* = .230). Post hoc analysis revealed that only the release of the abductor hallucis tendon resulted in a significant increase in coronal translation under 0-N PL (*P* = .033), whereas transection of the dorsal, plantar, or C1-C2 intercuneiform ligament did not produce significant changes. Under 10-N PL, none of the destabilization steps was associated with significant vertical displacement in the coronal plane, indicating effective compensation through PL activation (Table 3). The 2-way ANOVA confirmed a significant effect of PL force (*P* < .001), with post hoc comparison showing a significant difference between 0-N and 10-N PL only in the fully destabilized condition after sectioning of M. abductor hallucis and dorsal, plantar and C1-C2 intercuneiform ligament (cut 4) (Table 4).

**Table 3.** Sidak’s Multiple Comparisons Test for Vertical Traction of the First Metatarsal Head in the Coronal Plane (0- and 10-N Peroneus Longus [PL] Force Analyzed Separately).^a^

Comparison	0-N PL Force			10-N PL Force		
	Mean Difference ± SE (degrees)	Adjusted *P* Value	95% CI (degrees)	Mean Difference ± SE (degrees)	Adjusted *P* Value	95% CI (degrees)
Baseline vs cut 1	0.9 ± 0.21	.033*	−1.68 to −0.09	0.4 ± 0.29	.642	−1.5 to 0.7
Cut 1 vs 2	0.2 ± 0.58	.997	−2.37 to 2.0	0.3 ± 0.44	.960	−1.96 to 1.4
Cut 2 vs 3	0.1 ± 0.42	.995	−0.5 to 0.61	0.1 ± 0.18	.978	−0.58 to 0.77
Cut 3 vs 4	1.1 ± 0.37	.131	−2.49 to 0.34	0.7 ± 0.34	.359	−1.95 to 0.61

a Values are mean differences in angular translation (degrees) between destabilization levels ± SE. The 95% CIs relate to the mean difference between the 2 levels being compared and do not refer to the absolute mean translation at each level.

* Significance.

**Table 4.** Two-Way ANOVA and Sidak’s Multiple Comparisons Test for the Effect of Peroneus Longus (PL) Force During Vertical Traction of the First Metatarsal Head in the Coronal Plane.^a^

	0-N PL Force	10-N PL Force			
	Mean Translation (degrees)	Mean Translation (degrees)	Mean Difference ± SE (degrees)	Adjusted *P* Value	95% CI (degrees)
Baseline	0.5	0.01	0.5 ± 0.65	.748	−1.06 to 2.1
Cut 1	1.3	0.3	1.0 ± 0.65	.140	−0.57 to 2.59
Cut 2	1.5	0.6	0.9 ± 0.65	.213	−0.67 to 2.49
Cut 3	1.5	0.5	1.0 ± 0.65	.179	−0.63 to 2.53
Cut 4	2.6	1.2	1.4 ± 0.65	<.022*	−0.22 to 2.94

a The columns “0-N PL Force” and “10-N PL Force” show the mean angular translation (degrees) at each destabilization level. “Mean Difference ± SE” reports the mean difference in translation between 0-N and 10-N PL force (0 N minus 10 N) ± SE, and the 95% CIs relate to the mean difference rather than the CI of the absolute mean translation.

* Significance.

## Discussion

This biomechanical study analyzed the stabilizing structures preventing the first metatarsal from dorsal or medial deviation after TMT-I fusion, focusing on both passive ligamentous restraints and dynamic muscular stabilization. The main finding was that peroneus longus activation provides a significant dynamic stabilizing effect on the first ray, particularly in the transverse plane, where it can largely compensate for progressive ligamentous insufficiency. At the same time, the results underscore the critical passive stabilizing role of the dorsal and plantar tarsometatarsal ligaments and, in particular, the medial-middle (C1-C2) intercuneiform ligament, which together provide baseline resistance against medial drift that is further supported by PL activation.

Coronal stability of the first ray was shown to be predominantly maintained by the abductor hallucis, whereas the dorsal, plantar, and C1-C2 intercuneiform ligaments contribute only minimally to superior-inferior stabilization. This finding is particularly noteworthy given that the primary line of action of the abductor hallucis lies in the transverse plane. Despite this orientation, the tendon seems to provide substantial resistance against dorsal displacement of the first ray, suggesting that its dynamic stabilizing capacity exceeds that of the passive ligamentous restraints. Considering that the dorsal tarsometatarsal ligaments are regarded as the weakest components of the Lisfranc ligament complex, the abductor hallucis may play an important role in supporting dorsal stability of the first ray. Activation of the PL becomes relevant in the coronal plane only when all passive stabilizers have been removed.

In contrast, transverse stability is primarily provided by the dorsal and plantar Lisfranc ligaments and, most importantly, by the C1-C2 intercuneiform ligament, which constitute the key passive stabilizer against medial deviation, although the sequential sectioning design means an order effect on this finding cannot be excluded. In addition, PL activation exerts a strong dynamic stabilizing effect in the transverse plane and can compensate for ligamentous insufficiency even before all passive stabilizers have been disrupted. The stabilizing effect of PL activation on TMT-I is well known. Therefore, a preventive role in the pathogenesis of HV or recurrence of HV after surgical realignment has been hypothesized.^22^ In former biomechanical studies, the effect of PL force on first metatarsal alignment has been proven. In a similar experimental setup, Bohne et al^27^ found a stabilizing effect of PL in the transverse plane in cadaveric feet without TMT-I fusion. This is in concordance with our findings. Nevertheless, Bohne et al focused on the effect of PL, neglecting the impact of the dorsal and plantar Lisfranc and C1-C2 intercuneiform ligament. Additionally, only the transverse plane has been examined. Faber et al^28^ showed a stabilizing effect of PL in the coronal plane in cadaveric feet; no effect in the transverse plane was detected.

Both Bohne et al^27^ and Faber et al^28^ performed their experiments without weight bearing. The advantage of the current study is that it was performed under weight-bearing conditions with 200 N axial load, which more closely simulates physiological loading during the stance phase. Even though the model was static, the applied axial load generated compressive forces that counteracted tensile forces acting on the first ray and thereby reproduced essential aspects of in vivo load transmission. This approach may better reflect the interaction between dynamic muscular stabilizers and passive ligamentous restraints than non–weight-bearing models. An effect of weightbearing on foot morphology and first ray alignment has been shown in several studies.^17^ Also ligamentous instability is more apparent on weight-bearing radiographs.^30^ Furthermore, Bohne and Faber focused on TMT-I instability, whereas our study evaluated residual instability after TMT-I fusion. After TMT-I fusion, the lever arm for displacement is increased and stabilizing structures are not directly comparable.

In our study, a significant effect of PL force on preservation of MT-I alignment has been found both for the transverse and coronal planes, whereby the effect was more pronounced in the transverse plane. It was shown that PL function remains intact after TMT-I arthrodesis.^24^ Nevertheless, there is a recurrence rate of HV deformity after TMT-I arthrodesis of 3%, raising the question which structures could be insufficient, facilitating a recurrent malalignment. According to Fleming et al,^12^ a residual intercuneiform instability assessed by the “Hook-Test” can be found in nearly 80% of TMT-I fusions. After treatment with an additional medial-middle intercuneiform screw, he reported satisfactory results. To the author’s knowledge, studies comparing the recurrence rate of HV after exclusive TMT-I arthrodesis and after TMT-I arthrodesis with additional intermetatarsal or intercuneiform fixation are missing. In the study of Long et al,^30^ a high nonunion rate of 20% were found for intercuneiform and intermetatarsal fusions with increasing IMA in postoperative follow-ups indicating the stabilizing effect of these fixations leading to loss of realignment in case of nonunion. Recently, a widening of the medial-middle intercuneiform joint and its association with HV recurrence has been shown after modified Lapidus procedure in weight-bearing computed tomography. Involved structures have not been analyzed.^31^

Besides PL, the Lisfranc-ligament complex is crucial in the tarsometatarsal stabilization. The TMT-I joint as part of the Lisfranc-joint line is embedded in a complex mesh with dorsal, plantar, and interosseus ligaments.^32^ A post-traumatic development of HV deformity after Lisfranc injury has been described.^33^ Although separate stabilizing effects of PL and ligament structures are described in the literature, their simultaneous effect and the significance of passive ligamentous structures depending on PL activation has not been evaluated yet.

In the current study, an elementary stability of first ray alignment after TMT-I arthrodesis against transverse force is maintained by the ligamentous complex. Gradual sectioning of dorsal, plantar, and medial-middle intercuneiform ligaments significantly decreases transverse stability. PL was shown to compensate for this ligamentous instability, preventing a significant decrease of first ray stability in the transverse plane after ligamentous sectioning. In the coronal plane, the stabilizing effect of the ligaments was less distinctive, with no significant decrease in stability after gradual sectioning, whereas sectioning of the M. abductor hallucis tendon led to a significant decrease in coronal stability. PL force only had a significant effect after sectioning of dorsal, plantar, and also C1-C2 intercuneiform ligaments. Nevertheless, a tendency toward decreased dislocation in the coronal plane was noted when PL force was applied.

Overall, our findings show the importance of sufficient ligaments in the preservation of first ray alignment and support the concept that the medial-middle (C1-C2) intercuneiform ligament is a key passive restraint against medial drift of the first ray after TMT-I fusion. An increase in intercuneiform distance was associated with recurrence of HV deformity. The increased postoperative distance has been attributed to higher forces and transverse movements after TMT-I arthrodesis.^34^ Furthermore, patients with HV show higher stress loads on the intercuneiform joint, possibly resulting in intercuneiform instability due to degenerative changes in the ligamentous structures.^35^ Regarding the results of the current study, intraoperative accidental transection of the intercuneiform ligament also has to be considered. Therefore, performing the Hook-Test by Fleming et al after TMT-I stabilization seems to be reasonable to exclude initial instability. This experiment was deliberately designed as a biomechanical cadaver model to explore stabilizing mechanisms under progressively increasing ligamentous insufficiency and different levels of PL activation, rather than to reproduce any specific clinical constellation of hallux valgus recurrence. Accordingly, the findings are interpreted as mechanistic insight into first ray stability after TMT-I fusion and not as a direct answer to which exact structures are responsible for recurrence in vivo. Furthermore, the present model was designed to assess first ray stability after TMT-I fusion under defined loading of the first metatarsal and does not allow conclusions about generalized midfoot instability.

Nevertheless, although this study demonstrates that ligamentous insufficiency results in measurable biomechanical instability, the direct causal relationship between this laboratory-measured instability and clinical HV recurrence remains to be established. It is plausible that loss of ligamentous integrity contributes to recurrence, but clinical studies with long-term radiographic and functional follow-up are needed to confirm this hypothesis. In addition to peroneus longus, the tibialis anterior tendon may also contribute to first ray stability through its dorsiflexing and inverting effect on the medial column. In the present model, tibialis anterior loading was kept constant and its specific contribution was therefore not assessed separately. Future studies may further clarify the interaction between tibialis anterior and peroneus longus in stabilizing the fused first ray.

This study also has some limitations. First, a stabilizing effect of skin and tissue is eliminated after preparation of the specimens so that first ray instability is potentially more pronounced in this model. Second, differences in ligamentous stability might have biased the results. Because this was a cadaver study, a direct transmission to clinical implications is not possible. Additionally, because the ligaments were cut in a fixed sequential manner, the cumulative destabilization may have amplified the apparent contribution of later structures such as the medial-middle intercuneiform ligament. A further limitation is that we did not perform separate mechanical testing of the TMT-I fusion construct; therefore, we cannot completely exclude minor motion within the arthrodesis, although intraoperative fluoroscopy, visual inspection under axial loading, and the final control condition suggested sufficient construct stability for the purposes of this study. At least, this study was performed under static loading conditions with a constant axial load of 200 N. Dynamic changes in plantar contact area and pressure distribution, as seen during gait, were not simulated and may limit the transferability of the findings to physiological conditions. Because of the small sample size, formal testing for normality was not performed; thus, the use of parametric ANOVA relies on its robustness to moderate deviations from normality, and the results should be interpreted with appropriate caution.

Overall, this study complements current data to static and dynamic first ray stabilizers providing biomechanical insights into the interaction of PL and Lisfranc and medial-middle intercuneiform ligaments and can help clinicians decide whether to use an intercuneiform stabilizing screw or not. Whether an intercuneiform screw can completely compensate for loss of ligamentous stability needs to be evaluated in further studies.

## Conclusion

Dorsal and plantar tarsometatarsal ligaments and the medial-middle intercuneiform ligament act as important static stabilizers of transverse first ray alignment after TMT-I fusion in this cadaveric model, whereas the peroneus longus provided relevant dynamic stabilization. In the coronal plane, the abductor hallucis acted as the primary passive stabilizer; ligament sectioning produced no significant coronal instability, and peroneus longus compensation in this plane was significant only under full destabilization. Gradual sectioning of the ligamentous restraints increased transverse instability, which was largely compensated by peroneus longus activation under the experimental conditions. Given the small sample size and the simplified, sequentially destabilized model, these findings should be interpreted as exploratory biomechanical insight rather than definitive evidence of clinical behavior. Future clinical studies should build on existing weight-bearing computed tomography data on medial-middle intercuneiform alignment and clarify how postoperative intercuneiform widening relates to recurrent deformity, symptoms and functional outcome, and prospective trials of peroneus longus strengthening or targeted orthotic concepts aimed at supporting PL function may help to determine whether dynamic stabilization of the first ray can reduce recurrence rates after TMT-I fusion.

## Supplemental Material

sj-pdf-1-fai-10.1177_10711007261460458 – Supplemental material for Passive and Dynamic Stabilizers of First Ray Alignment After TMT-I Fusion: The C1-C2 Intercuneiform Ligament and Peroneus Longus in a Sequential Cadaveric Destabilization Model Supplemental material, sj-pdf-1-fai-10.1177_10711007261460458 for Passive and Dynamic Stabilizers of First Ray Alignment After TMT-I Fusion: The C1-C2 Intercuneiform Ligament and Peroneus Longus in a Sequential Cadaveric Destabilization Model by Alena Richter, Peter Savov, Sarah Ettinger, Leif Claassen, Daiwei Yao, Frederik Emanuel Mann, Anna Altemeier and Christian Plaass in Foot & Ankle International
