# Supplementary material for: Passive and Dynamic Stabilizers of First Ray Alignment After TMT-I Fusion: The C1-C2 Intercuneiform Ligament and Peroneus Longus in a Sequential Cadaveric Destabilization Model
Source: Foot Ankle Int. 2026 Jul 21;47(9):1207–16. doi: 10.1177/10711007261460458 (PMC13589112; doi:10.1177/10711007261460458)
Supplement: Supplementary file 1 [file sj-pdf-1-fai-10.1177_10711007261460458.pdf]

FAI and FAO Disclosure Form

**Author Name:**	**Alena Richter**
**Manuscript Title:**	**Stabilizing structures of the first tarsometatarsal joint after isolated TMT I-arthrodesis in the treatment of hallux valgus – a cadaver study**
**Manuscript Number:**	Click or tap here to enter text.
**Date:**	**08.12.2025**

1. List any project funding, consulting fees, material support (including surgical supplies, cadavers, or the use of any products from related companies) or any other form of assistance valued over $500 received in the past three years.

Items relevant to this specific manuscript: specify type (consultancies, royalties, research support, other support, intellectual property etc.)

Not applicable

2. Identify any additional financial or non-financial relationships from the past three years that could be perceived as conflicts of interest or could introduce bias in your submitted work (consultancies, royalties, research support, other support, intellectual property etc.)

Not applicable

This form will be available to the readership by publication as an online supplement to the article. Items relevant to this specific manuscript will be listed within the article text.

FAI and FAO Disclosure Form

**Author Name:**	**Peter Savov**
**Manuscript Title:**	**Stabilizing structures of the first tarsometatarsal joint after isolated TMT I-arthrodesis in the treatment of hallux valgus – a cadaver study**
**Manuscript Number:**	Click or tap here to enter text.
**Date:**	**08.12.2025**

1. List any project funding, consulting fees, material support (including surgical supplies, cadavers, or the use of any products from related companies) or any other form of assistance valued over $500 received in the past three years.

Items relevant to this specific manuscript: specify type (consultancies, royalties, research support, other support, intellectual property etc.)

None.

2. Identify any additional financial or non-financial relationships from the past three years that could be perceived as conflicts of interest or could introduce bias in your submitted work (consultancies, royalties, research support, other support, intellectual property etc.)

Consultant for Smith&Nephew and Microport

This form will be available to the readership by publication as an online supplement to the article. Items relevant to this specific manuscript will be listed within the article text.

FAI and FAO Disclosure Form

**Author Name:**	**Sarah Ettinger**
**Manuscript Title:**	**Stabilizing structures of the first tarsometatarsal joint after isolated TMT I-arthrodesis in the treatment of hallux valgus – a cadaver study**
**Manuscript Number:**	Click or tap here to enter text.
**Date:**	**08.12.2025**

1. List any project funding, consulting fees, material support (including surgical supplies, cadavers, or the use of any products from related companies) or any other form of assistance valued over $500 received in the past three years.

Items relevant to this specific manuscript: specify type (consultancies, royalties, research support, other support, intellectual property etc.)

none

2. Identify any additional financial or non-financial relationships from the past three years that could be perceived as conflicts of interest or could introduce bias in your submitted work (consultancies, royalties, research support, other support, intellectual property etc.)

none

This form will be available to the readership by publication as an online supplement to the article. Items relevant to this specific manuscript will be listed within the article text.

FAI and FAO Disclosure Form

Author Name: Leif Claassen
Manuscript Title: Stabilizing structures of the first tarsometatarsal joint after isolated TMT I-arthrodesis in the treatment of hallux valgus – a cadaver study
Manuscript Number: Click or tap here to enter text.
Date: 08.12.2025

1. List any project funding, consulting fees, material support (including surgical supplies, cadavers, or the use of any products from related companies) or any other form of assistance valued over $500 received in the past three years.

   Items relevant to this specific manuscript: specify type (consultancies, royalties, research support, other support, intellectual property etc.)

   /

2. Identify any additional financial or non-financial relationships from the past three years that could be perceived as conflicts of interest or could introduce bias in your submitted work (consultancies, royalties, research support, other support, intellectual property etc.)

   /

This form will be available to the readership by publication as an online supplement to the article. Items relevant to this specific manuscript will be listed within the article text.

FAI and FAO Disclosure Form

**Author Name:**	**Daiwei Yao**
**Manuscript Title:**	**Stabilizing structures of the first tarsometatarsal joint after isolated TMT I-arthrodesis in the treatment of hallux valgus – a cadaver study**
**Manuscript Number:**	Click or tap here to enter text.
**Date:**	**08.12.2025**

1. List any project funding, consulting fees, material support (including surgical supplies, cadavers, or the use of any products from related companies) or any other form of assistance valued over $500 received in the past three years.

Items relevant to this specific manuscript: specify type (consultancies, royalties, research support, other support, intellectual property etc.)

none

2. Identify any additional financial or non-financial relationships from the past three years that could be perceived as conflicts of interest or could introduce bias in your submitted work (consultancies, royalties, research support, other support, intellectual property etc.)

none

This form will be available to the readership by publication as an online supplement to the article. Items relevant to this specific manuscript will be listed within the article text.

FAI and FAO Disclosure Form

**Author Name:**	**Frederik Mann**
**Manuscript Title:**	**Stabilizing structures of the first tarsometatarsal joint after isolated TMT I-arthrodesis in the treatment of hallux valgus – a cadaver study**
**Manuscript Number:**	Click or tap here to enter text.
**Date:**	**08.12.2025**

1. List any project funding, consulting fees, material support (including surgical supplies, cadavers, or the use of any products from related companies) or any other form of assistance valued over $500 received in the past three years.

Items relevant to this specific manuscript: specify type (consultancies, royalties, research support, other support, intellectual property etc.)

The authors declare that no project funding, consulting fees, material support, or other assistance related to this manuscript was received in the past three years

2. Identify any additional financial or non-financial relationships from the past three years that could be perceived as conflicts of interest or could introduce bias in your submitted work (consultancies, royalties, research support, other support, intellectual property etc.)

The authors declare no additional financial or non-financial relationships that could be perceived as a conflict of interest

This form will be available to the readership by publication as an online supplement to the article. Items relevant to this specific manuscript will be listed within the article text.

FAI and FAO Disclosure Form

**Author Name:**	**Anna Altemeier**
**Manuscript Title:**	**Stabilizing structures of the first tarsometatarsal joint after isolated TMT I-arthrodesis in the treatment of hallux valgus – a cadaver study**
**Manuscript Number:**	Click or tap here to enter text.
**Date:**	**08.12.2025**

1. List any project funding, consulting fees, material support (including surgical supplies, cadavers, or the use of any products from related companies) or any other form of assistance valued over $500 received in the past three years.

Items relevant to this specific manuscript: specify type (consultancies, royalties, research support, other support, intellectual property etc.)

-

2. Identify any additional financial or non-financial relationships from the past three years that could be perceived as conflicts of interest or could introduce bias in your submitted work (consultancies, royalties, research support, other support, intellectual property etc.)

-

This form will be available to the readership by publication as an online supplement to the article. Items relevant to this specific manuscript will be listed within the article text.

FAI and FAO Disclosure Form

**Author Name:**	**Christian Plaasse**
**Manuscript Title:**	**Stabilizing structures of the first tarsometatarsal joint after isolated TMT I-arthrodesis in the treatment of hallux valgus – a cadaver study**
**Manuscript Number:**	Click or tap here to enter text.
**Date:**	**08.12.2025**

1. List any project funding, consulting fees, material support (including surgical supplies, cadavers, or the use of any products from related companies) or any other form of assistance valued over $500 received in the past three years.

Items relevant to this specific manuscript: specify type (consultancies, royalties, research support, other support, intellectual property etc.)

Medartis (consultant, paid speaker), IBRA (research support, consultancies,

2. Identify any additional financial or non-financial relationships from the past three years that could be perceived as conflicts of interest or could introduce bias in your submitted work (consultancies, royalties, research support, other support, intellectual property etc.)

Novastep (consultanie, speaker)
NewClip (consultancies, speakers)

This form will be available to the readership by publication as an online supplement to the article. Items relevant to this specific manuscript will be listed within the article text.
